# A Submucosal Moderately Poor Differentiated Laryngeal Squamous Cell Carcinoma Presenting as a Thyroid Mass: A Report of a New Case

**DOI:** 10.1155/crot/6231543

**Published:** 2025-05-28

**Authors:** Zhihan Liu, Meng Lei, Ruimin Zhao, Xiaobao Yao, Yanxia Bai, Shaoqiang Zhang

**Affiliations:** Department of Otorhinolaryngology and Head Neck Surgery, The First Affiliated Hospital of Xi'an Jiaotong University, Xi'an 710061, Shaanxi, China

**Keywords:** diagnostic points, SCC, SLC, treatment

## Abstract

**Background:** Submucosal laryngeal carcinoma (SLC) is a rare subtype of transglottic laryngeal carcinoma (TLC) and usually originates from the center of the laryngeal ventricle. Nearly 70% of TLC cases are confined to the larynx and 30% show external laryngeal infiltrations. Early stage asymptomatic TLC usually shows occult lesions and tends to grow into lateral parapharyngeal space.

**Case Summary:** We present an unusual case of submucosal moderately poor differentiated laryngeal squamous cell carcinoma (SCC) characterized by thyroid mass, hoarseness, and dyspnea as the main symptoms. In this case, a 60-year-old Chinese male was made a preliminary diagnosis of suspected thyroid carcinoma (TC) with laryngeal metastases to the cricoid cartilage. No laryngeal neoplasms were observed under nasopharyngo-fiberoscope. After giving 1-month therapy with anlotinib, the tumor lesion had minimal response while the patient insisted on surgical section to relieve dyspnea. Intraoperative frozen section biopsy confirmed that the tumor was moderately poor differentiated SCC of the larynx, and in this case, thyroid metastases might result in SLC penetration of cricothyroid membrane.

**Conclusion:** SLC should be taken into consideration in the case of suspicious TC with laryngeal cartilage infiltration and subglottic area infiltration. Further coarse needle puncture or surgical biopsy should be carried out to clarify diagnosis to optimize treatment strategy.

## 1. Introduction

Generally, laryngeal carcinoma (LC) can be divided into 3 major types, including supraglottic, glottic, and subglottic carcinoma according to the anatomic location. Transglottic laryngeal carcinoma (TLC), also known as parapharyngeal space laryngeal cancer, is a rare subtype of LC, while the definition of TLC remains controversial and unclear. Mcgavran et al. defined TLC as tumors that cross the laryngeal ventricles (LVs) and invade both the supraglottic and glottic areas [1]. In contrast, Cao et al. defined TLC as tumors located in the center of the LV that longitudinally invade adjacent regions [2]. This variability in definitions leads to inconsistencies in diagnosis and treatment strategies, making it challenging for clinicians to agree on a standardized approach. The controversy surrounding the definition of TLC significantly impacts patient management. Precise identification of TLC based on specific anatomical characteristics can influence decisions regarding surgery or radiation therapy. Misclassification of TLC may lead to inappropriate treatment plans, resulting in suboptimal patient outcomes.

Mostly, the first and major clinical complaint of TLC is hoarseness caused by unilateral vocal cord fixation, and laryngeal surface mucosal irregularities can be seen on the nasopharyngo-fiberoscope. Nearly 70% of TLC confined to the larynx and 30% showed extra laryngeal infiltrations [3]. Also, the rarer LC that originates in the submucosa of the larynx called submucosal laryngeal carcinoma (SLC) is often classified as a special type of TLC because it often straddles the supraglottic and glottic regions and has an unclear site of locating [4]. Early stage asymptomatic SLC usually shows occult lesions and tends to grow into lateral parapharyngeal space. Then, pharyngodynia could appear as the tumor expands into the parapharyngeal space and destroys the laryngeal cartilages. Irritating dry coughs, caused by SLC invasion to at least one side of the laminae of the thyroid cartilage and cricothyroid membrane, could occur occasionally. In some rare cases, the tumor extends directly into the thyroid through the glottis, anterior commissure, anterior angle of the thyroid cartilage, gaps between tracheal rings, or cricothyroid membrane, causing secondary carcinoma of the thyroid [5, 6].

To enhance diagnostic accuracy, refining the definitions of TLC and SLC may be beneficial. This could involve integrating imaging modalities and histopathological criteria. Establishing clear margins for what constitutes TLC could streamline referral pathways and facilitate preoperative planning. Once a consensus is reached regarding the definition, tailored treatment approaches can be developed, taking into account tumor behavior, including the propensity for local invasion or metastasis, which may improve management strategies.

Here reports a submucosal moderately poor differentiated laryngeal squamous cell carcinoma (SCC) characterized by thyroid mass as the main symptom and diffusely invaded to the deep connective tissue, skeletal muscle, thyroid tissue, and cervical lymph nodes. To our knowledge, few cases have been reported in the literature so far. Similar published reports and literature reviews were also reviewed on this rare disease.

## 2. Report of the Case

**Figure 1:**
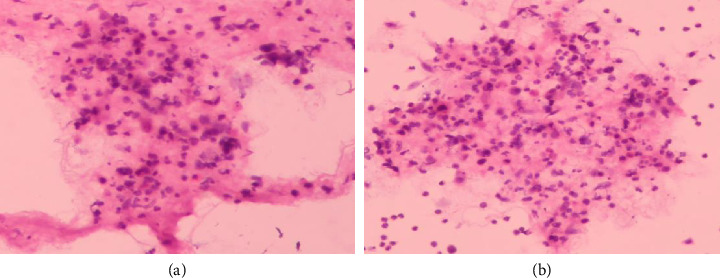
Preoperative microscopic characteristics of thyroid fine-needle aspiration.

**Figure 2:**
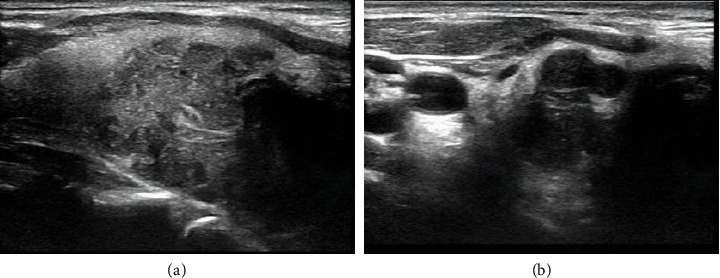
Preoperative ultrasound images of neck. (a) The image showed a large hypoechoic nodule on the right lobe of thyroid. (b) The image showed a lot of multiple circular and hypoechoic abnormal cervical lymph nodes.

**Figure 3:**
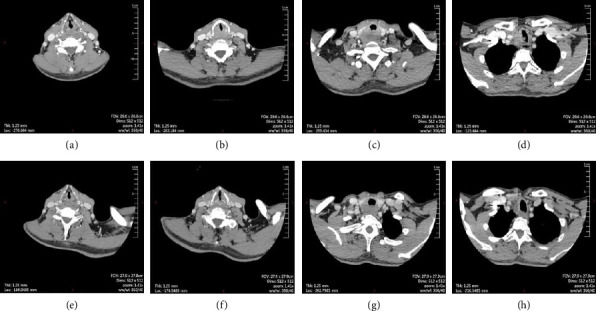
Preoperative enhanced computed tomography axial images of the neck. (a–d) The images showed that the patient had abnormally enhanced right lobe before the start of targeted therapy, indicating TC, tumor lesions range of about 30 × 43 × 50 cm, the left lobe of the thyroid gland, ring cartilage, trachea, and subglottic areas had been invaded, and bilateral multiple cervical lymphadenectasis. (e–h) The images showed that the tumor lesion area of the patient had been reduced to 34 × 36 × 48 cm after 1 month of targeted therapy, and the density was reduced compared with the previous time.

**Figure 4:**
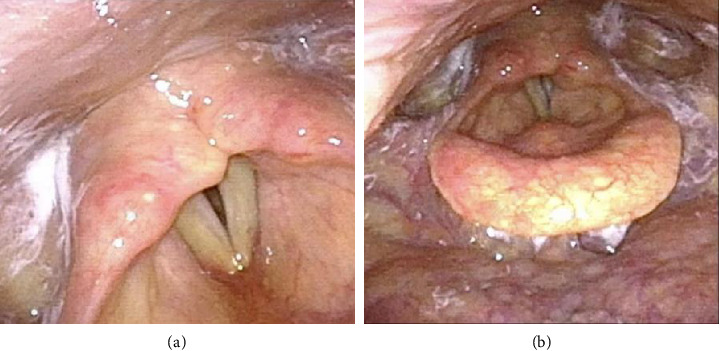
Preoperative fiberlaryngoscopic images of larynx. (a) The image showed the fixation of the right vocal cord and the inactivity of left vocal cord, which closed with a small gap. (b) The image showed that the pyriform fossa was symmetrical bilaterally, the mucosa was smooth, and no neoplasm was found, as well as the epiglottis mucosa was smooth, and the motor function was normal.

A 60-year-old Chinese male complained of progressive hoarseness and dyspnea for more than 3 months in our department. He had already done the ultrasound of the neck and fine-needle aspiration (FNA) of the thyroid nodule in a local clinic, and the initial pathological diagnosis was papillary thyroid carcinoma (TC). The pathologists at our hospital did not find the follicular epithelial papillary structures typical of papillary TC during consultation. Thus, the second pathologic diagnosis suggests suspicious papillary TC or metastatic cancer of another tissue origin (Figure 1). The ultrasound of the neck suggested that there was a large hypoechoic TI-RADS 5 nodule around 24 × 26 × 33 mm on the right side of the thyroid involving the trachea and dorsal envelope. Also, multiple circular and hypoechoic abnormal cervical lymph nodes were observed from both bilateral VI and VII areas and right-side IV areas (Figures 2(a) and 2(b)). The CT confirmed an abnormal enhancement at the right lobe of the thyroid, which hinted at TC. The extent of the tumor lesion was about 30 × 43 × 50 cm, where the left lobe of the thyroid, annular cartilage, trachea, and subglottic area had been violated. Bilateral cervical lymph nodes hyperplasia were present at the same time (Figures 3(a), 3(b), 3(c), and 3(d)). The nasopharyngo-fiberoscope merely showed the fixation of the right vocal cord and the inactivity of the left vocal cord (Figures 4(a) and 4(b)). Laboratory testing revealed an elevated thyroid-stimulating hormone (TSH) level of 8.86 uIU/mL (normal range: 0.3–4.5 uIU/mL). Therefore, we preliminary diagnosed as suspected papillary TC with laryngeal metastases.

To reduce the progression of thyroid lesions and endeavor to perform a total R0/R1 thyroidectomy to preserve the larynx, 1-month therapy of anlotinib was empirically given to the patient by the initial clinician, anlotinib is a novel tyrosine kinase inhibitor that has shown great antitumor activity and tolerability in several types of TC [7–9], while the thyroid lesion area was insignificantly response to 34 × 36 × 48 cm in the reexamined CT (Figures 3(e), 3(f), 3(g), and 3(h)). The patient insisted on surgical treatment to relieve dyspnea as soon as possible.

Prior medical history was hypertension for 6 years, chronic hepatitis C with cirrhosis of hepatic decompensation for 6 months, and smoking history for 30 years, an average of 20 cigarettes per day.

## 3. Treatment

After admission, the patient undertook a detailed physical examination and related preoperative examinations. First, a tracheotomy was conducted to keep the unobstructed of the respiratory tract. Since cases of TC metastasis to the larynx and LC metastasis to the thyroid were both rare, the primary site and pathological type of the tumor remained unclear, and an intraoperative frozen section biopsy of thyroid tumor tissue was operated which confirmed the presence of cancerous tissue, probably SCC, in the right lobe and isthmus of the thyroid. Therefore, during the operation, it was suspected that the primary tumor came from tissues other than the thyroid. When carefully searched along the growth path of thyroid cartilage infiltrating tumors and finally traced them to the LV, we found a large number of morphologically abnormal tissues in the submucosa of the larynx and confirmed the larynx as the primary tumor site. Thus, the patient subsequently underwent a total laryngectomy, total thyroidectomy, functional right lymph node excision, and functional bilateral VI and VII levels lymph node excision under general anesthesia to reduce the risk of recurrence.

**Figure 5:**
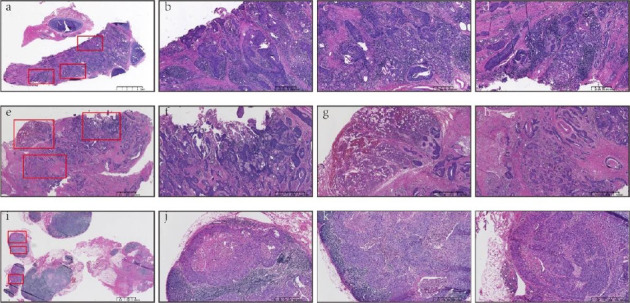
Postoperative final histopathology of the resected specimen. (a–d) The images showed a submucosal invasive moderately poorly differentiated SCC of the larynx with lymphovascular invasion. (e–h) The images showed the tumor invasion and metastasis in thyroid tissue, which was consistent with the source of laryngeal carcinoma. (i–l) The images showed the tumor invasion and metastasis in lymph nodes on the side of the right neck, which was consistent with the source of laryngeal carcinoma (hematoxylin and eosin-stained specimen).

The final histopathology of the resected specimen suggested an infiltrating moderately poorly differentiated SCC of the larynx with lymphovascular invasion, where the tumor was 0.3 mm from the left incisal margin. Moreover, the tumor had already encroached on deep connective tissue, skeletal muscles, and thyroid cartilages, as well as infiltrated and transferred to the thyroid (left-right lobes and isthmus), a small piece of the parathyroid (near the right lobe), and trachea. Numerous lymph node metastases had occurred, including 10/17 paratracheal lymph nodes, 1/1 lymph node adjacent to the isthmus, 1/1 lymph node adjacent to the left lobe of the thyroid, and 2/10 lymph nodes from right cervical clearance. Immunohistochemistry showed the tumor stained positively with PD-L1 (Sp263 antibody, CPS: +10; TPS: +1%) (Figures 5(a), 5(b), 5(c), 5(d), 5(e), 5(f), 5(g), 5(h), 5(i), 5(j), 5(k), and 5(l)).

Based on these pathology reports, we confirmed the diagnosis as “submucosal invasive moderately poorly differentiated SCC of the larynx (SLC, pT4aN2M0).”

After 5 months of the surgery, this patient began to receive radiotherapy, chemotherapy, and immunotherapy.

## 4. Discussion

SLC is a special type of TLC that is clinically rare and more prone to misdiagnosis when associated with external laryngeal invasion. The main reasons for this include the lack of obvious characteristic clinical manifestations, unclear locations of submucosal lesions, the extensive range of advanced lesions, and difficulties in pathological diagnosis. These factors make the diagnosis and differential diagnosis of SLC extremely challenging in clinical practice [10]. According to the eighth edition of the American Joint Committee on Cancer's Tumor Node Metastasis (TNM) classification, LC is staged as T4a when it involves the thyroid. The overall survival (OS) of patients with advanced laryngeal SCC who underwent total laryngectomy is approximately 57.5 months, which is the preferred treatment for T4 laryngeal SCC [11, 12]. The high misdiagnosis rate of SLC associated with external laryngeal invasion can lead to inadequate treatment strategies, indicating the need for increased vigilance and improved diagnostic procedures in clinical settings. This report aims to summarize the main diagnostic points of SLC with external extension, clarify the diseases that require differential diagnosis, and explore potentially effective treatment strategies, ultimately providing better management plans for future patients with this rare condition.

### 4.1. History and Risk Factors

A significant risk factor for laryngeal SCC is tobacco use, which has a recognized correlation with laryngeal cancer [13]. However, interestingly, some studies suggest a negative correlation with the risk of TC, indicating the complexity of these relationships [14–16].

### 4.2. Clinical Symptoms and Metastatic Patterns

The incidence of LC invading the thyroid is roughly 1%∼30%, and its probability in TLC is 2/15 [17]. SLC invades the thyroid in three ways: (1) directly through thyroid cartilage or cricothyroid membrane; (2) remetastasis via lymphatic vessels to anterior laryngeal lymph nodes or deep cervical lymph nodes; and (3) metastasis through vasculum. Brennan et al. found that 78% of thyroid invasion of laryngeal SCC are through the first route and 10% through the second route [18]. The most common modality of direct tumor infiltration includes (a) deep submucosal infiltration (52%); (b) deep submucosal infiltration with mucosal surface infiltration (38%); and (c) infiltration along the mucosal surface (10%). SLC extends to the supraglottic and glottic regions, mainly in (a) mode [2]. Furthermore, Nayak et al. found that moderately differentiated tumors have a propensity for indirect spread [19]. However, tracheal invasion in well-differentiated TC is uncommon with approximately 1%–13% incidence [20]. Lymphatic metastases occur in approximately 30%–80% of papillary TC, which could directly infiltrate the larynx and trachea [21, 22]. The clinical symptoms of SLC and TC diseases are quite similar, that is, hoarseness and dyspnea are often present. Usually, hoarseness is first complained of when the tumor originates in the larynx, and when the tumor originates in the thyroid, a thyroid nodule is first complained of, followed by a feeling of pressure in neck. Both may be accompanied by varying degrees of dyspnea, especially if LC invades the vocal cords or a thyroid mass compresses the trachea.

### 4.3. Imaging and Endoscopy

CT, MRI, PET, ultrasound, and other imaging examinations can provide valuable anatomic space assessment, supplement clinical information on tumor volume and extrapharyngeal invasion, assist in surgical plan making, and determine whether the primary tumor is resectable. Endoscopy can provide information about tissue structure noninvasively (Table 1) [23–27]. Cartilage infiltration on laryngeal CT is characterized by sclerosis, erosion, lysis, and transmural [28]. MRI appears to be more sensitive than CT in detecting cartilage infiltration, which should be suspected if the cartilage exhibits signal strength similar to that of a tumor [29]. External laryngeal infiltration usually presents as substitution by tumor tissue on the outside of the membrane or cartilage or fat loss of external laryngeal structures (such as blood vessels or muscles) and laryngeal components on CT. External laryngeal infiltration should be considered when the intensity of the signal in the fat component of the external laryngeal soft tissue on MRI that is continuous with the primary tumor and the intensity of laryngeal infiltration is similar to that of the tumor on T2-weighted images and is hypointense on T1-weighted images [23].

### 4.4. FNA and Immunostaining

The overall incidence of secondary carcinoma of the thyroid ranges from 1.2% to 24%, and the incidence of thyroid SCC caused by invasion of submucosal squamous cell LC into the adjacent thyroid is even less common. Therefore, misdiagnosis is easy to occur when using FNA to diagnose thyroid SCC. This is partly because thyroid SCC must be cytologically distinguished from a variety of pathological types, such as squamous metaplasia, papillary TC or undifferentiated TC, squamous differentiation, and mucoepidermoid carcinoma. On the other hand, tumors secondary to the thyroid gland are usually multifocal, so there is a certain probability that there is no component of SCC in the selection of the location during FNA needle aspiration [30–33]. Thyroid SCC cells are microscopically heterogeneous and may contain patchy, clustered, or scattered malignant squamous cells, moderate anaplastic cells, deep staining of nuclei, squamous cell nests in the cytoplasm, keratinized beads, and intercellular bridges. Papillary TC cells usually present a typical follicular epithelial papillary structure, with nuclei having nuclear sulcus, intranuclear inclusion bodies, nuclear overlaps, and ground-glass nuclei. Undifferentiated TC cells exhibit pleomorphism and nuclear division, with massive necrosis, neovascularization, and inflammatory cell infiltration in tumor tissue, which manifests as sarcomatous, epidermoid, or squamous changes. Due to the cytological polymorphisms of thyroid SCC, the misdiagnosis rate of diagnosis using FNA alone is extremely high, so an additional diagnostic tool, immunohistochemistry, is needed for further differential diagnosis. Thyroid SCC is often positive for cytokeratin 5/6, cytokeratin 19, and p53; Papillary TC often expressed significant thyroglobulin (TG) and thyroid transcription factor (TTF). Undifferentiated TC diffusely expressed p63 and Paired Box 8 (PAX8) [33–35]. Although FNA cytology can help us correctly diagnose thyroid SCC, it is not possible to determine whether it is a primary or secondary lesion. The secondary SCC of the thyroid is a diagnosis of exclusion, and more and more accurate tumor-related information is needed.

### 4.5. Treatment

Most cases of SLC are diagnosed at advanced stages, making total laryngectomy accompanied by postoperative radiation therapy the standard treatment approach. Outcomes following total laryngectomy and chemoradiation have shown variable disease-free survival rates, indicating the ongoing challenges and need for innovation in treatment strategies [36].

Although clinical cases related to SLC are rare, we have made efforts to search for other reports associated with SLC and highlighted significant diagnostic and treatment challenges through comparative analysis. Variations in diagnostic accuracy, disease progression rates, and treatment outcomes emphasize the necessity of raising awareness and standardizing protocols in the clinical management of SLC. By synthesizing the findings from different studies, our clinicians can gain deeper insights into the deficiencies of current diagnostic and treatment strategies for SLC and identify areas that require further exploration and clarification, ultimately improving the diagnostic accuracy and treatment efficacy for patients with this challenging condition.

## 5. Conclusion

In conclusion, it is essential to consider SLC in cases where there is a suspicion of TC involving laryngeal cartilage infiltration and subglottic area involvement. This is particularly true for patients presenting with hoarseness and dyspnea, especially those with a significant history of smoking. Imaging studies that reveal tumor infiltration in the thyroid, laryngeal cartilage, and subglottic region should prompt further investigation. The rarity of SLC adds a layer of complexity to the diagnostic process, especially given the potential for misclassification between primary and secondary tumors, which can significantly influence treatment decisions and prognostic outcomes. Recent research underscores the ongoing controversies surrounding the definitions of SLC and its relationship to TLC, highlighting the need for standardized criteria that incorporate both clinical and pathological findings. By addressing these definitions and their implications, we can refine our approach to diagnosis and management, ultimately improving patient care and outcomes in those affected by this challenging and often overlooked malignancy.

## Figures and Tables

**Table 1:** Imaging for laryngeal carcinoma (LC).

Imaging projects	Main uses and imaging traits	Advantages	Disadvantages
Computed tomography (CT)	1. Primary tumor (PT): compare the degree of enhancement between tumor and normal head and neck structure 2. Cartilage infiltration (CI): shape of the cartilage and depth of invasion 3. Lymph node involvement: evaluate the pathological lymph node 4. External laryngeal spread (ELS): evaluate the tumor tissue replacement outside of the membrane or cartilage	1. Better than MRI for the evaluation of bony anatomy 2. Easily identify tumor growth outside the larynx	Impossible to distinguish between nonossified cartilage and tumor in the larynx

MR imaging (MRI)	1. PT: identify disease involvement of the laryngeal ventricle and characterize the involvement of the anterior commissure 2. CI: cartilage shows similar signal intensity to the tumor 3. ELS: identify extra laryngeal soft tissue fat contiguous with the PT	1. Greater soft tissue definition, especially at the parapharyngeal space and thyroid 2. Detect small areas of marrow space invasion	Exhibits some false positive features similar to CI

Ultrasound	1. PT: identify epiglottis, vocal cords, anterior commissure, and subglottic 2. CI: evaluate the pathological lymph node 3. ELS: identify thyroid	Determine the number of head and neck lymph node transfers	Calcification of thyroid cartilage does not achieve effective ultrasound penetration

White light endoscopy (WLE)	1. Laryngoscopy: assess the gross appearance and growth pattern (exophytic vs. submucosal) 2. Bronchoscopy: examination of areas that are difficult to assess (pharynx, LV, and lining of the trachea)	Indirect mirror laryngoscopy (IML): give an excellent overview of the tongue base and provide excellent color and depth perception Flexible fiber-optic laryngoscopy (FFL): assess vocal fold movement and movement of the cricoarytenoid joint and visualize the subglottic with topical anesthesia	IML: difficult to visualize the anterior commissure, especially in patients with a strong gag reflex FFL: sometimes do not distinguish mucosal differences Bronchoscopy: difficulty in detecting airway lesions located distal

Abbreviations: CI, cartilage infiltration; CT, computed tomography; ELS, external laryngeal spread; FFL, flexible fiber-optic laryngoscopy; IML, indirect mirror laryngoscopy; MRI, MR imaging; PT, primary tumor; WLE, white light endoscopy.

## Data Availability

Data sharing is not applicable to this article as no datasets were generated or analyzed during the current study.

## Nomenclature

SLC: Submucosal laryngeal carcinoma
TLC: Transglottic laryngeal carcinoma
SCC: Squamous cell carcinoma
TC: Thyroid carcinoma
LC: Laryngeal carcinoma
LV: Laryngeal ventricles
TNM: Tumor node metastasis
OS: Overall survival
FNA: Fine-needle aspiration
TSH: Thyroid-stimulating hormone
TG: Thyroglobulin
TTF: Thyroid transcription factor
CT: Computed tomography
MRI: MR imaging
WLE: White light endoscopy
PT: Primary tumor
CI: Cartilage infiltration
ELS: External laryngeal spread
IML: Indirect mirror laryngoscopy
FFL: Flexible fiber-optic laryngoscopy
